# Evidence of Submicroscopic *Plasmodium knowlesi* Mono-Infection in Remote Indigenous Communities in Kelantan, Peninsular Malaysia

**DOI:** 10.4269/ajtmh.23-0184

**Published:** 2023-09-25

**Authors:** Nor Diyana Dian, Ahmad Basyir Muhammad, Elora Nor Azman, Nur Ashrina Eddie, Nur Iman Azmi, Valerie Chong Tze Yee, Mohd Amirul Fitri A. Rahim, Mohd Bakhtiar Munajat, Mohd Ikhwan Mukmin Seri Rakna, Muhd Rafiq Mohd Kasri, Ahmad Imran Mohamed, Nuraffini Ghazali, Noor Wanie Hassan, Siti Nor Azreen Abdul Manap, Emelia Osman, Wathiqah Wahid, Sriwipa Chuangchaiya, Inke Nadia D. Lubis, Paul C. S. Divis, Sherwin Chan, Zulkarnain Md Idris

**Affiliations:** ^1^ Department of Parasitology and Medical Entomology, Faculty of Medicine, Universiti Kebangsaan Malaysia, Kuala Lumpur, Malaysia;; ^2^ District Health Office of Gua Musang, Kelantan, Malaysia;; ^3^ Department of Community Health, Faculty of Public Health, Kasetsart University, Chalermphrakiat Sakon Nakhon Province Campus, Sakon Nakhon, Thailand;; ^4^ Department of Paediatric, Faculty of Medicine, Universitas Sumatera Utara, Medan, Indonesia;; ^5^ Malaria Research Centre, Faculty of Medicine and Health Sciences, Universiti Malaysia Sarawak, Sarawak , Malaysia;; ^6^ Department of Microbiology, Tumor and Cell Biology, Karolinska Institutet, Stockholm, Sweden

## Abstract

Malaysia has maintained zero cases of indigenous human malaria since 2018. However, zoonotic malaria is still prevalent in underdeveloped areas and hard-to-reach populations. This study aimed to determine the prevalence of malaria among remote indigenous communities in Peninsular Malaysia. A cross-sectional survey was conducted in six settlements in Kelantan state, from June to October 2019. Blood samples were tested for malaria using microscopy and nested polymerase chain reaction (nPCR) targeting the *Plasmodium* cytochrome c oxidase subunit III (*cox3*) gene. Of the 1,954 individuals who appeared healthy, no malaria parasites were found using microscopy. However, nPCR revealed seven cases of *Plasmodium knowlesi* mono-infection (0.4%), and six out of seven infections were in the group of 19 to 40 years old (*P* = 0.026). No human malaria species were detected by nPCR. Analysis of the DNA sequences also showed high similarity that reflects common ancestry to other *P. knowlesi* isolates. These findings indicate low submicroscopic *P. knowlesi* infections among indigenous communities in Malaysia, requiring PCR-based surveillance to support malaria control activities in the country.

Malaria is a life-threatening infectious disease caused by parasites transmitted through *Anopheles* mosquito bites. Five species of the *Plasmodium* parasite cause malaria in humans, with *Plasmodium falciparum* being the most lethal. In 2020, about 241 million people in 85 malaria-endemic countries were affected by malaria, resulting in 627,000 deaths globally.^1^ The WHO Western Pacific Region, which includes Malaysia, had less than 1% of this burden.^1^ While Malaysia has achieved zero indigenous human malaria cases since 2018 due to successful control measures, zoonotic malaria caused by *Plasmodium knowlesi* remains a public health concern, particularly in underdeveloped areas including remote regions of Malaysian Borneo and among indigenous populations in Peninsular Malaysia.^2^

In 2021, Malaysia reported 3,575 *P. knowlesi* cases, resulting in 13 deaths.^3^ The Orang Asli, Peninsular Malaysia’s indigenous people, continue to be at high risk of malaria infection due to their remote settlements in forested areas, where they are more susceptible to mosquito bites and potential exposure to the parasite through monkey reservoir hosts and mosquito vectors.^4^^,^^5^ Despite significant investment and effort, access to diagnosis and treatment of indigenous populations in remote communities in Malaysia remains inconsistent due to logistical and communication challenges, extreme weather, and terrain conditions. Knowledge gaps also exist regarding malaria infection and endemicity among indigenous communities in Peninsular Malaysia. Thus, we aimed to determine the prevalence of malaria infections among indigenous Orang Asli communities in Kelantan state, with emphasis on *P. knowlesi*.

A cross-sectional survey with a convenience sampling strategy was carried out between June and October 2019. Six Orang Asli settlements were surveyed, namely Kuala Betis (4°53′22″N, 101°45′30″E), Mendrop (4°40′28.5″N, 101°33′28.9″E), Gob (5°25′00″N, 101°65′82″E), Bihai (4°52′60″N, 101°58′01″E), Tuel (4°46′10″N, 101°28′09″E) and Brooke (4°67′41″N, 101°48′94″E) in Gua Musang district, Kelantan state, Peninsular Malaysia (Figure 1). The study was conducted in accordance with the Declaration of Helsinki and was approved by the Medical Ethics Committee of the National University of Malaysia (no. UKM PPI/111/8/JEP-2019-148). Respondents were sensitized to the study objectives and procedures by the local health district personnel for the study participation. Written informed consent was obtained from all study participants and/or guardians before enrolment. All community members who had general good health, willingness to provide samples, and had consented were included in the study. Participants who were treated for malaria within the past four weeks or those presently on treatment for malaria were not included. Participants not willing to participate and/or had not signed the informed consent were excluded from the study. The participant’s history of experiencing any symptoms of malaria, age, gender and location of the settlement were recorded at the time of enrolment.

**Figure 1.**
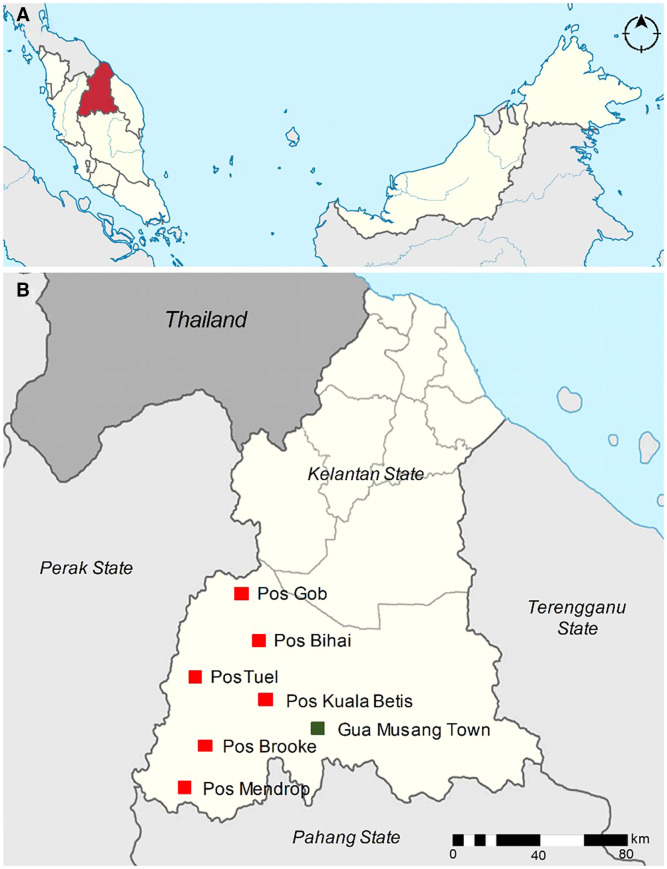
Map of study location. (**A**) Map of Peninsular Malaysia showing the state of Kelantan (red). (**B**) Study location of six settlements in Gua Musang district, Kelantan.

Thin and thick blood smears were prepared on site, stored in slide boxes and transported daily to the main laboratory in Gua Musang, where thin blood smears were fixed with methanol. All smears were stained with 3% Giemsa solution (Merck, Darmstadt, Germany) for 30 minutes and examined under oil emersion (10 × 100 magnification) by experienced microscopists. For malaria detection by nested polymerase chain reaction (nPCR), blood samples withdrawn by finger prick using BD Microtainer Contact-Activated Lancet (Becton Dickinson, Franklin Lakes, NJ) were spotted on Whatman ET31 Chr filter papers (Whatman International, Maidstone, UK) and dried thoroughly at ambient temperature. The dried blood spots were put in individual zipped plastic bags and stored at −20°C. DNA was extracted from three 3-mm filter paper punches using the QIAamp Blood Mini Kit (Qiagen, Germantown, MD) according to the manufacturer’s instructions. *Plasmodium* infection (primary PCR) and species identification (secondary PCR) were conducted following a previously published method targeting the *Plasmodium* mitochondrial cytochrome c oxidase subunit III (*cox3*) gene,^6^ with newly designed primer sets of species-specific for *Plasmodium vivax* (MtNst_vivF2: 5′-TATTATTGTCTATACTAGATACTATAGTT-3′ and MtNst_vivR: 5′-GTTCTTTTTCTATTCAGAATAATGAATATAT-3′) and *P. knowlesi* (MtNst_knoF: 5′- CTTAATTGTCTATACTAGATACTATGGAC-3′ and MtNst_vivR: 5′- GTTCTTTTTCTATTCAGAATAATGAATAT-3′). Species-specific primers for the secondary PCR for *P. falciparum*, *Plasmodium malariae*, and *Plasmodium ovale* were as described previously.^6^ The amplified products of positive samples by nPCR were subsequently sent to BioNuclix Sdn. Bhd. (Kuala Lumpur, Malaysia) for direct DNA sequencing to validate the identities of the species.

All data were analyzed using Microsoft Excel software and double-checked to avoid errors. Then, the data were processed and analyzed using statistical software STATA/SE version 13.1 (StataCorp, College Station, TX). χ^2^ statistical test or Fisher’s exact test were conducted to study the relationship between malaria infection and sociodemographic variables such as gender, age group and location of the settlement. The DNA sequences generated were confirmed for *Plasmodium* DNA sequence using the Basic Local Alignment Search Tool (BLAST) from the National Center for Biotechnology Information (NCBI) website.

A total of 1,954 individuals participated in this study (Table 1). Microscopic examination of Giemsa-stained thick and thin blood films showed no individual positive with malaria parasites. Nevertheless, molecular screening of dried blood samples by nPCR protocol detected 7 (0.4%) *P. knowlesi*, and none were positive for other malaria species. Of the 7 *P. knowlesi*-positive individuals, 5 (71.4%) were males, with no significant difference in malaria-specific prevalence observed between gender (*P* = 0.251). Furthermore, *P. knowlesi* prevalence differed significantly by age group (*P* < 0.001): highest in the 19 to 40 group (0.8%), followed by ≤ 18 (0.1%) and no parasite was detected in the > 40 group by nPCR. *Plasmodium knowlesi* cases were also observed in 2 out of 6 settlements, namely Brooke (*n =* 5) and Mendrop (*n =* 2). Furthermore, the *cox3* gene of malaria parasites from seven *P. knowlesi*-positive isolates were successfully amplified and sequenced. Analysis of the DNA sequences based on BLAST from the NCBI database revealed high sequence similarity that reflects common ancestry to other *P. knowlesi* isolates for all seven samples. The pairwise identity ranges from 99.31% to 99.77% (Table 2).

**Table 1:** Prevalence of submicroscopic malaria infections among the indigenous Orang Asli populations in Gua Musang, Kelantan in 2019

Characteristic	No. of individuals	Malaria positive by microscopy	Nested PCR assay		Proportion malaria positive by PCR (%)	*P* value†
			*Plasmodium knowlesi*	Other *Plasmodium* spp.*		
Overall	1,954	0	7	0	0.4	–
Gender‡						
Male	840	0	5	0	0.6	0.251
Female	1,104	0	2	0	0.2	
Age group						
≤ 18	941	0	1	0	0.1	0.026
19–40	714	0	6	0	0.8	
> 40	289	0	0	0	0.0	
Settlement						
Kuala Betis	640	0	0	0	0.0	–
Mendrop	193	0	2	0	1.0	
Gob	180	0	0	0	0.0	
Bihai	164	0	0	0	0.0	
Tuel	146	0	0	0	0.0	
Brooke	631	0	5	0	0.8	

* Nested PCR assays for *Plasmodium falciparum*, *Plasmodium vivax*, *Plasmodium malariae* and *Plasmodium ovale*.

† Comparing the proportion of *P. knowlesi* positive by PCR between sub-categories.

‡ Total of 10 individuals with no data on gender and age.

**Table 2:** BLAST search of *cox3* sequences of *Plasmodium* spp.

Sample ID	Percent identity (%)	*Plasmodium* species reference
P018	99.50	*Plasmodium knowlesi* isolate EU880498
P026	99.77	*P. knowlesi* isolate AY598141
B202	99.31	*P. knowlesi* isolate LR701176
B204	99.55	*P. knowlesi* isolate LT727662
B687	99.32	*P. knowlesi* isolate KU245038
B711	99.65	*P. knowlesi* isolate KJ569858
B724	99.66	*P. knowlesi* isolate AB444108

BLAST = Basic Local Alignment Search Tool. All seven positive samples confirmed *P. knowlesi* with a high percentage of identity with the simian *Plasmodium* nucleotide sequences published in the GeneBank database.

This study is the first to report malaria epidemiological data by active case surveillance among remote Orang Asli communities in Malaysia since the country declared zero indigenous human malaria cases in 2018. The present study also highlights the prevalence of submicroscopic malaria infections with exclusive attention on *P. knowlesi* malaria. The collected data shows that the submicroscopic prevalence of *P. knowlesi* mono-infection in the study areas in Kelantan was 0.4%, highlighting the importance of molecular detection in malaria surveillance and the alarming evidence of zoonotic malaria infection among the hard-to-reach population in Malaysia, particularly the Orang Asli.

The majority of cases were submicroscopic *P. knowlesi* mono-infection and could not be detected by conventional malaria parasite microscopy. Unlike previously reported submicroscopic infections among Orang Asli in Malaysia,^7^ all the infections were not associated with coinfection involving other malaria species. Nevertheless, results from the present study are consistent with previous studies where *P. knowlesi* accounted for the majority of the positive cases.^8^ These variable observations demonstrate that the parasites causing these infections may be at or below the level of detection for assays developed for use primarily on clinical samples.^9^ Therefore, the use of microscopy as the sole diagnostic method likely leads to an underestimation of the *P. knowlesi* malaria burden.^10^^,^^11^ Furthermore, analysis of relatively small volumes of blood, such as from dried blood spots collected from individuals with very low-density infections, means that parasites may be missed even by repeat molecular assays.^12^ Requirement for better-optimized molecular assays is needed to understand the true burden of malaria prevalence and avoid ineffective interventions in the affected community as well as the wider population.

Higher proportions of *P. knowlesi* were observed in the 19 to 40 age group of the Orang Asli communities. Previous studies among indigenous communities in Sabah have also reported that *P. knowlesi* infection occurs more commonly in adults than in children.^13^^,^^14^ As a working-age population, adults Orang Asli are responsible for their family economy and thus strive to gain additional income through forest activities such as foraging wild fruits, ornamental plants and wood products, and hunting wild animals.^4^ In addition, some of them stay longer in the forest to increase their earnings, especially during harvesting periods.^5^ This behavior increases the risk of *P. knowlesi* malaria among Orang Asli. Furthermore, local variation in *P. knowlesi* prevalence was also observed among Orang Asli communities in the study area. The present study showed that *P. knowlesi* cases were detected in only 2 (i.e., Brooke and Mendrop) out of 6 settlements. This finding is consistent with the clustering of human settlements similar to in Malaysia Borneo,^15^^–^^17^ which indicates the *P. knowlesi* infection came from peri-domestic exposure of competent specific *Anopheles* vector.^18^ One explanation for this is that the two settlements are located in the most southern part of Kelantan state and connected to each other by an access road, suggesting that human movement is another contributing factor to the micro-geographical variation in *P. knowlesi* endemicity. A recent study on land use change along the Sungai Kelantan Basin in Brooke settlement showed that the expansion of deforestation for logging and agricultural cropland has significantly impacted the temperature variations in the area.^19^ Consequently, temperature imbalance from deforestation may influence the functioning and stability of the natural ecological system (e.g., changes in mosquito oviposition sites and wildlife habitat), thus potentially increasing the threat of *P. knowlesi* malaria transmission and other zoonotic disease outbreaks.^2^^,^^3^

Our study has two limitations. Firstly, we only examined a limited number of sociodemographic variables among participants. Secondly, although our BLAST searches suggested possible matches, we were unable to conclusively establish species validation through phylogenetic analysis of the sequencing data. Despite these limitations, our study provides new insights into the prevalence of submicroscopic *P. knowlesi* infection among Orang Asli communities in Malaysia. We found a high proportion of *P. knowlesi* infections, particularly among adult Orang Asli, to be a significant endemic disease with potential health implications in the country. To effectively detect the infection, highly optimized and sensitive molecular methods are required. A guideline on the importance of submicroscopic *P. knowlesi* infections and a treatment policy is necessary to improve strategies for knowlesi malaria control in Malaysia.
